# Grating-based phase-contrast computed tomography for breast tissue at an inverse compton source

**DOI:** 10.1038/s41598-024-77346-1

**Published:** 2024-10-26

**Authors:** Daniel Berthe, Lisa Heck, Sandra Resch, Martin Dierolf, Johannes Brantl, Benedikt Günther, Christian Petrich, Klaus Achterhold, Franz Pfeiffer, Susanne Grandl, Karin Hellerhoff, Julia Herzen

**Affiliations:** 1 Chair of Biomedical Physics, Department of Physics, TUM School of Natural Sciences, Technical University of Munich, Garching, 85748 Germany; 2 Munich Institute of Biomedical Engineering, Technical University of Munich, Garching, 85748 Germany; 3 Research Group Biomedical Imaging Physics, Department of Physics, TUM School of Natural Sciences, Technical University of Munich, Garching, 85748 Germany; 4 Radiology Department, Red Cross Hospital, Munich, Germany; 5 TUM Institute for Advanced Study, Technical University of Munich, Garching, 85748 Germany; 6 Department of Diagnostic and Interventional Radiology, TUM School of Medicine, Klinikum rechts der Isar, Technical University of Munich, München, 81675 Germany

**Keywords:** Breast cancer, X-rays

## Abstract

The introduction of mammography screening programs has significantly reduced breast cancer mortality rates. Nevertheless, some lesions remain undetected, especially in dense breast tissue. Studies have shown that phase-contrast imaging can improve breast cancer diagnosis by increasing soft tissue contrast. Furthermore, grating-based phase-contrast imaging enables the simultaneous acquisition of absorption, phase-contrast, and scattering, so-called dark-field images. The latter allows the classification of microcalcifications. In addition, breast computed tomography (BCT) systems can identify and discriminate overlapping but clinically relevant structures. This study investigates the benefit of combining grating-based phase-contrast with BCT. We explore the potential of grating-based phase-contrast breast computed tomography (gbpc-BCT) with a breast phantom and a freshly dissected fibroadenoma. Improved image contrast could be achieved with radiation doses comparable to those used in clinical BCT.

## Introduction

Globally, breast cancer is the most prevalent cancer type among women. In 2018, an estimated 2.1 million new cases and $627\,000$ deaths were reported, accounting for $18\%$of all cancer deaths in women^1^. Early detection through mammography screening programs can reduce breast cancer mortality by up to $49\%$^2–4^. However, differentiation between benign lesions (i.e., cysts or fibroadenomas) remains a challenge leading to recall for additional imaging like ultrasound examination^5^. This is especially a problem for women with dense breast tissue who already have a significantly higher risk of developing breast cancer^6^.

A dedicated absorption-based photon counting BCT, which has been commercially available since recent years, could overcome the problem of overlapping structures by providing three-dimensional visualization of the breast at a radiation dose similar to standard mammography^7,8^. Initial results proved its potential in detecting suspicious masses^9^. Another advantage of BCT is its increased comfort over mammography^8^. Nevertheless, the challenge imposed by low soft-tissue contrast remains in absorption-based BCT. This can result in poor image contrast and often requires additional contrast agents^10^.

Phase-contrast imaging improves soft-tissue contrast without the administration of contrast agents. Unlike attenuation-based X-ray imaging, phase-contrast imaging utilizes the phase information of X-rays by measuring not only attenuation but also the refraction and the small-angle scattering signal induced by the specimen^11^. Various methods for obtaining the phase information exist, among others, propagation-based phase-contrast imaging^12–15^, Talbot-Lau interferometry^16,17^, edge illumination^18^and coherent diffractive imaging^19^. However, most of these methods, except for the Talbot-Lau interferometer, require a large synchrotron facility or a complex and expensive liquid metal jet X-ray source and can accordingly not be used in a clinical setting. Therefore, the preferred method for potential clinical use is the Talbot-Lau interferometer-based phase-contrast setup, which is used in this work.

Several studies have demonstrated the advantages of grating-based phase-contrast imaging, particularly its contrast enhancement, in two-dimensional mammography^20–22^. The additional phase information and the dark-field image show a diagnostic value by detecting the morphology of microcalcifications as a sign of early tumor malignancy^23–25^. Here, the performance of a gbpc-BCT is investigated. Its advantages could be twofold compared to mammography: It could discriminate clinically relevant structures overlapping in projections while improving soft-tissue contrast. Raupach et al^26^. already showed that especially a BCT, which operates at a high spatial resolution, may benefit from the additional phase information in terms of reduced patient dose due to the enhanced soft tissue contrast.

Two modern Photon counting detector (PCD)s, which deliver the required resolution and show a high quantum efficiency in the relevant energy range^27^, were used to perform a gbpc-BCT at a quasi-monochromatic inverse Compton X-ray source, followed by a comparison of the resulting attenuation and phase-contrast images.

Our study demonstrates that the phase image of a gbpc-BCT significantly enhances visual contrast andcontrast-to-noise ratio (CNR) compared to attenuation-based images. We have shown this by measuring a breast phantom and a freshly dissected fibroadenoma using a laboratory gbpc-BCT setup. The results lay the foundation for further investigation of a gbpc-BCT at a polychromatic X-ray source.

## Materials and methods

### Detectors

The two used detectors were the SANTIS GaAs 0808 HR ($75\,\upmu \hbox {m}$pixel size)^28^ and SANTIS GaAs 0804 ME (${150}\,\upmu \hbox {m}$pixel size)^29^ PCDs from DECTRIS Ltd. (Baden, Switzerland). Both detectors are equipped with a ${500}\,\upmu \hbox {m}$thick gallium arsenide sensor layer. The SANTIS GaAs 0808 HR has two adjustable energy thresholds^28^, and the SANTIS GaAs 0804 ME has four^29^. The used GaAs sensor layer has high quantum efficiency, especially at low photon energies up to ${50}\,\hbox {KeV}$which are relevant for mammography, making it ideal for the presented measurements^27,30^. In addition the PCDs provide a higher resolution compared to the commonly used flat panel detector, which is also crucial for reducing the patient dose^26^. For the conducted measurement, the images of the SANTIS GaAs 0808 HR were binned twice, resulting in the same pixel size of ${150}\,\upmu \hbox {m}$ for both detectors.

### Image acquisition at the MuCLS

**Fig. 1:**
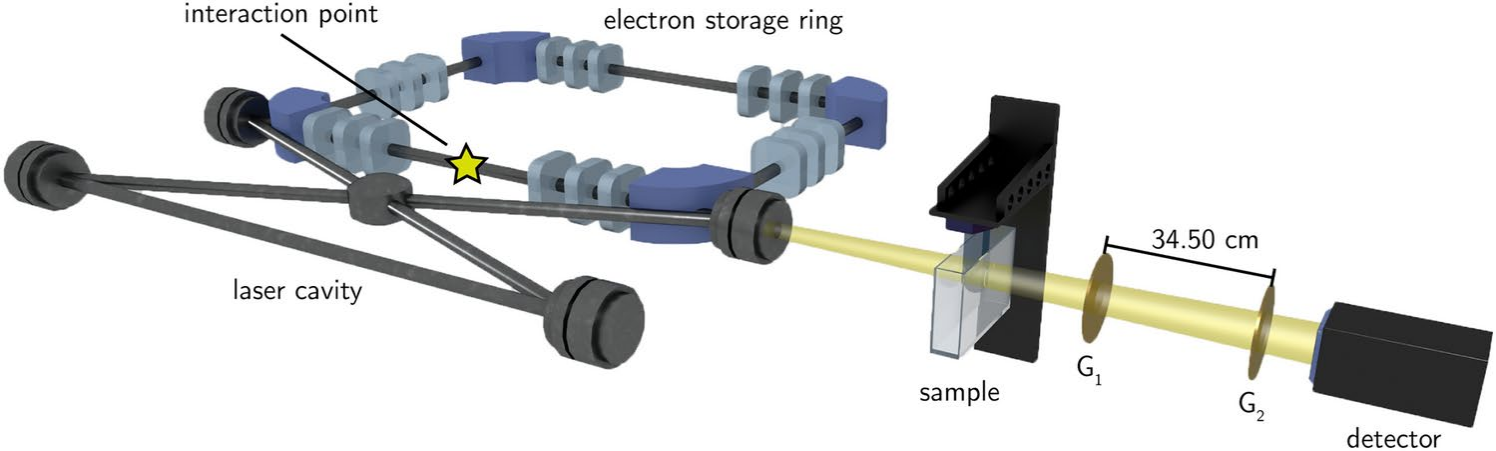
Scheme of the experimental setup. On the left, the Compact Light Source, consisting of an electron storage ring and a laser cavity, is sketched. X-ray photons are generated at the interaction point and illuminate the sample ${14.93}\,\hbox {m}$ downstream. Behind the sample, a Talbot-Lau grating interferometer and the detector are installed. Please note, that the proportions are not to scale.

The measurements were conducted at the Munich Compact Light Source (MuCLS) (cf. Fig. 1). The setup consists of an inverse Compton source (Lyncean Technologies Inc., Fremont, USA) that produces a highly brilliant, spatially coherent, and quasi-monoenergetic X-ray beam^31^and a dedicated imaging beamline, developed and installed by the Technical University of Munich, featuring two measuring hutches^32^ .

The sample was mounted on a rotation stage ${14.93}\,\hbox {m}$ downstream of the interaction point in a ${30}\,\hbox {mm}$ thick water container to avoid phase-wrapping. The Talbot-Lau grating interferometer consisting of a phase grating G_1_ and an analyzer grating G_2_ was set to an inter-grating distance $d_\text {G}$ of ${34.50}\,\hbox {cm}$^11^. The periods of the phase and analyzer grating were ${4.9}\,\upmu \hbox {m}$ and ${5.0}\,\upmu \hbox {m}$, respectively. The measurements were conducted at an X-ray energy of ${35}\,\hbox {KeV}$ with a visibility of $35\%$. A movable detector stage was positioned ${16.05}\,\hbox {m}$ downstream of the interaction point and directly behind the analyzer grating, which resulted in an effective pixel size of ${139.6}\,{\mu }\hbox {m}$. The acquisition parameters for the measurements are listed in Tab. 1. The samples were measured using a step-and-shoot approach. This means that for each projection angle, the analyzer grating was moved in five steps over the distance of one grating period, with an image taken at each position^33^. The resulting sinusoidal intensity curve for each pixel is called stepping-curve.

**Table 1:** Acquisition parameters.

Sample	Detector	Eff. pixel size	Exp. Time	Reconstruction Algorithm	Angles	Mean glandular dose
Breast Phantom	SANTIS GaAs 0808 HR	139.6	40	FBP	650	41
					163	10
Fibroadenoma	SANTIS GaAs 0804 ME	139.6	60	SIR	400	26
					140	9

### Processing

An expectation-maximization algorithm retrieves the attenuation and phase information from the sinusoidal stepping curve for all pixels^34^.

Especially for applications such as BCT, the dose for each projection is very low, which presents a challenge in accurately extracting the phase and attenuation information from the stepping curve. One method to improve image quality is to bin multiple pixels, which reduces noise but lowers image resolution. We applied patchwise phase retrieval, which reduces the noise without reducing the number of pixels but at the expense of image sharpness. It relies on the assumption that the sample does not contain sharp edges or other abrupt spatial changes. The assumption allows using the surrounding pixels as extra information in the expectation-maximization algorithm for each central pixel. This means we can calculate the phase and attenuation value for a three-by-three pixel patch and assign the resulting value to the central pixel in the patch.

That improves the accuracy of the fit for the signal retrieval, resulting in less noise in the final attenuation and phase-contrast images^35^. Afterwards, the projections were reconstructed using a Filtered back projection (FBP) or Statistical iterative reconstruction (SIR)^36^.

### Radiation dose

The applied Mean glandular dose (MGD) was calculated by the SIERRA Monte Carlo simulation-based model developed by Boone et al^37^. The model was originally developed for mammography. When it comes to BCT, the dose is calculated for a single projection and then multiplied by the number of frames. It takes tabulated monoenergetic normalized glandular dose coefficients ($\text {DgN}$), which depend on the photon energy *E*, glandularity *g*, and compressed breast thickness *t*, and the incident air Kerma *K* into account to calculate the MGD for arbitrary spectra:

1 $$\begin{aligned} \text {MGD} = \sum _{E=E_{min}}^{E_{max}} K \left( E\right) \text {DgN} \left( E, t, g \right) \kappa . \end{aligned}$$

Here, $\kappa$ denotes a conversion factor from air kerma to exposure. The DgN values are tabulated for a glandularity of $0\%$, $50\%$ and $100\%$. In the following, a glandularity of $50\%$ was assumed. All samples completely filled the Falcon tubes which they were measured in, allowing the tube diameter to be used directly as the sample thickness for the MGD calculation. Furthermore, the X-ray spectrum was measured using an energy-dispersive detector (Amptek X-123, Amptek Inc., USA).

### Samples

**Fig. 2:**
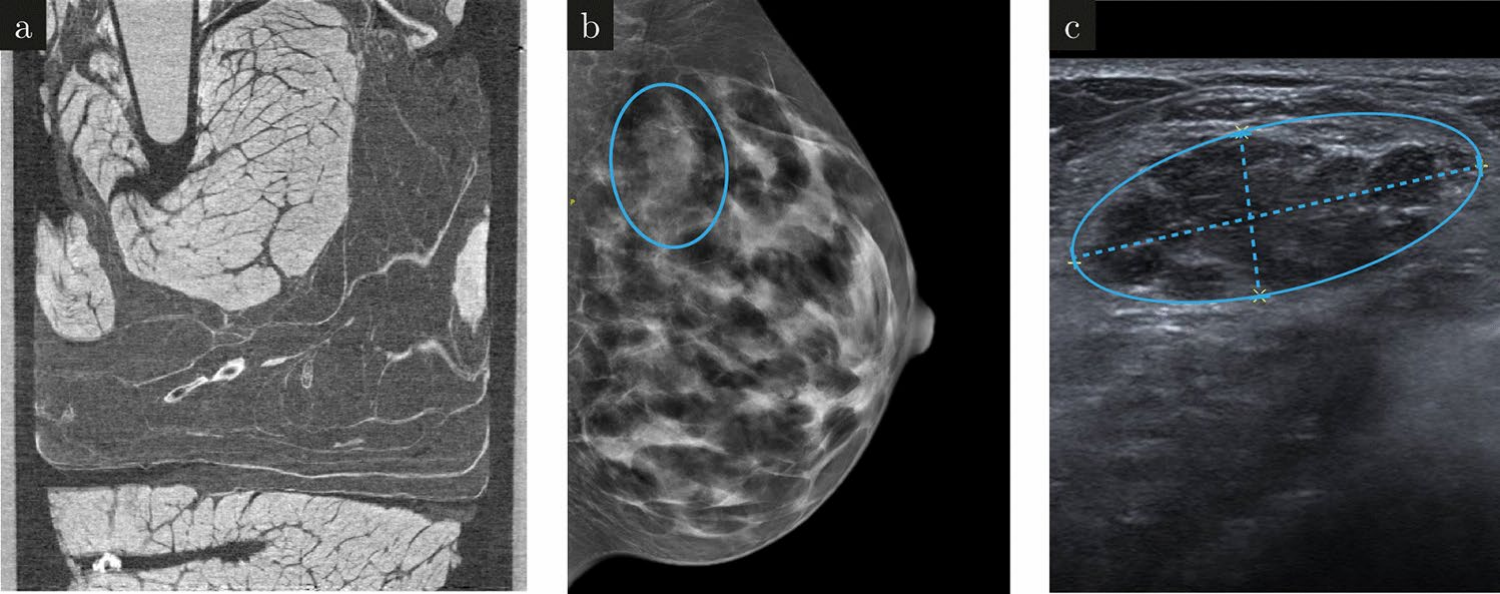
Sample images. A sagittal slice of a high dose phase-contrast CT of the breast phantom with all the added features is shown in (**a**). Prior to the surgical removal of the fibroadenoma, a mammography image was taken. The fibroadenoma shows up as a bright structure in the mammography image (**b**) which correlates to a dark region in the ultra sound image (**c**).

The study was conducted under the Declaration of Helsinki and approved by the local ethics committee (Ethik-Kommission der Bayerischen Landesärztekammer (BLAEK), number 19063, date of permission 30/09/2019), and informed consent was obtained from the patient. For the conducted gbpc-BCT measurements, a breast-like phantom made from pork neck and a freshly dissected fibroadenoma provided by the Red Cross Hospital Munich were used. The breast phantom consists of a fatty piece of pork to mimic the adipose breast tissue (cf. Fig. 2(a)). It was fixated in a $4\%$ formaldehyde solution before being immersed into a Falcon tube containing a $70\%$ ethanol solution. An Eppendorf tube filled with a $6\,\hbox {mg/mL}$iodine solution (IMERON 400 MCT, Bracco Imaging Deutschland GmbH) was added. The iodine concentration mimics a realistic contrast agent concentration in a patient’s blood vessels^38^. A polymethyl methacrylate (PMMA) rod was added for a possible energy calibration.

Clinical mammography and ultrasound examination were performed before the surgical removal of the fibroadenoma. The mammography (Fig. 2b) revealed a ${3.6}\,\hbox {cm}$ lesion marked with a blue circle. Although the lesion is also visible on ultrasound (Fig. 2c), further Core-needle biopsy was required to differentiate between a benign fibroadenoma and a malignant carcinoma. After surgical removal, the fibroadenoma was embedded in a $4\%$ formaldehyde solution for fixation, and a PMMA rod for energy calibration was added.

## Results

**Fig. 3:**
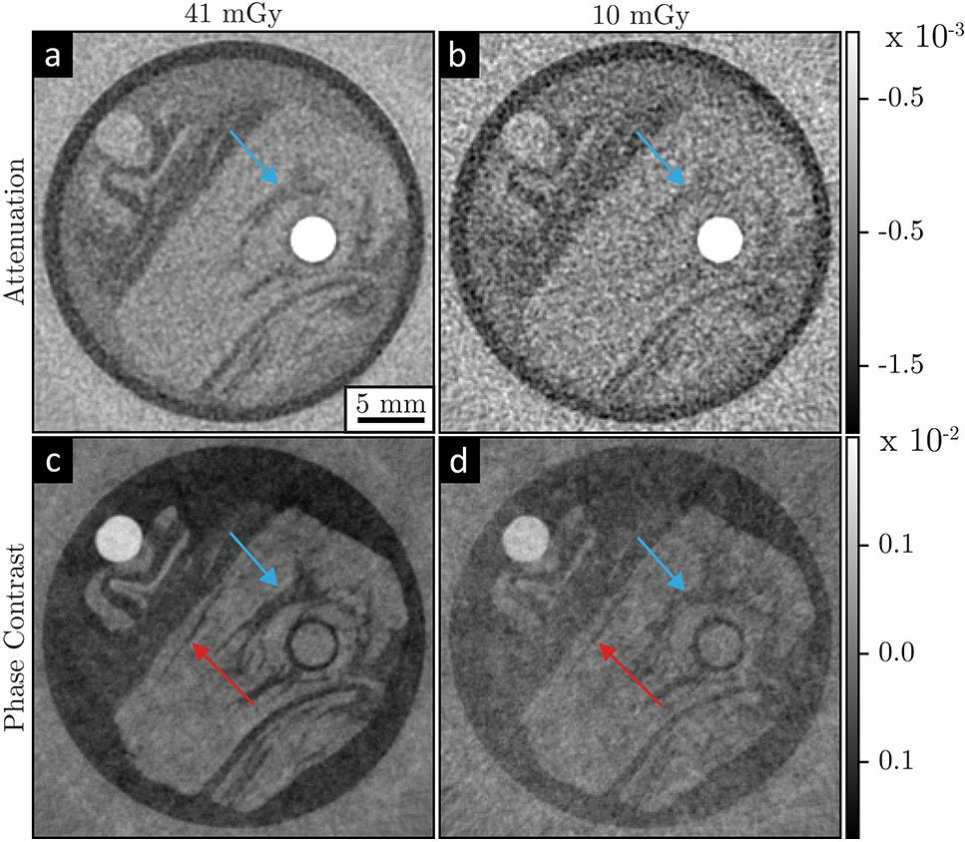
Low dose gbpc-BCT with a breast phantom. Axial attenuation-contrast (**a**, **b**) and phase-contrast (**c**, **d**) reconstructions measured with a MGD of ${41}\,\hbox {mGy}$ in the first column and ${10}\,\hbox {mGy}$ in the second column, respectively. The arrows mark regions, where the contrast enhancement of gbpc-BCT is particularly evident.

As a first proof of principle, a gbpc-BCT with a breast phantom was conducted with the measurement parameters listed in Table 1. Figure 3 shows the reconstructed axial slices of the attenuation (a, b) and phase-contrast images (c, d). These images were all extracted from the same scan, but with different numbers of projections to modify the dose. The first column (a, c) shows the results obtained with a higher dose of ${41}\,\hbox {mGy}$, while the low-dose images displayed in the second column (b, d) are achieved with a dose of ${10}\,\hbox {mGy}$.

The reconstructions show visually a superior contrast of the phase-contrast images compared to the attenuation-contrast images at both radiation doses. This is particularly evident in the region marked with a blue arrow where the dark adipose tissue is indistinguishable from the muscle tissue in the low-dose attenuation image but is visible in the phase-contrast image. Furthermore, the thin structure indicated by the red arrow in the phase-contrast images can still be seen, even in the image with a reduced dose, while it is not visible in the higher or lower dose attenuation-contrast images. The attenuation and phase images are extracted from the same scan with gratings in the beam, which results in almost twice the radiation dose compared to a standard BCT, due to the additional analyser grating between the sample and the detector.

**Fig. 4:**
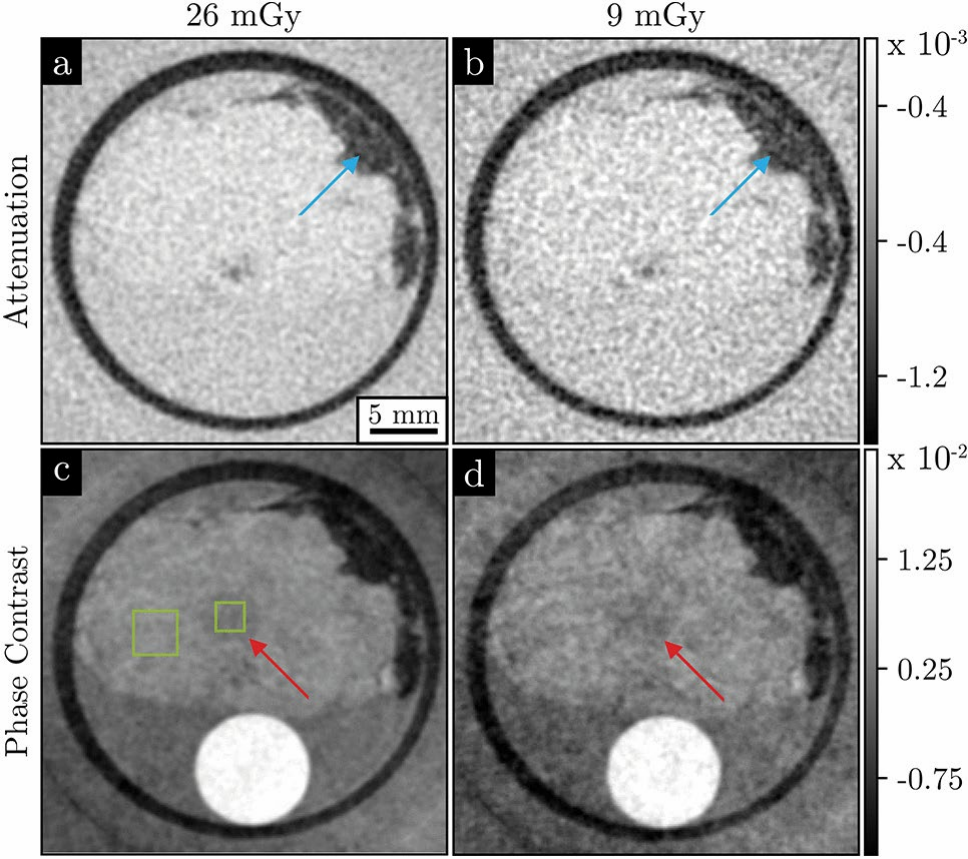
Dose-dependent comparison of the fibroadenoma’s attenuation and phase-contrast images. The images were taken at a dose of ${26}\,\hbox {mGy}$ (**a**, **c**) and ${9}\,\hbox {mGy}$ (**b**, **d**). The first row shows the attenuation-contrast images and the second row depicts the phase-contrast images extracted from the same measurement. Adipose tissue, indicated by the blue arrow, exhibits a strong contrast in both image modalities, whereas low contrast structures, indicated by the red arrow, can only be identified in the phase-contrast images (**c**) and (**d**). The green boxes in (**c**) mark the sample regions used for the calculation of the CNR.

For a more realistic investigation of gbpc-BCT, a freshly dissected fibroadenoma was scanned. Figure 4 shows an axial slice of the reconstructed attenuation (a, b) and phase-contrast images (c, d) of the fibroadenoma. The first column depicts a high-dose measurement at ${26}\,\hbox {mGy}$, which is used as a ground truth for the low-dose measurement in the second column at ${9}\,\hbox {mGy}$. The lower dose was achieved by reducing the number of angles used for the reconstruction.

In the attenuation-contrast images in Fig. 4(a, b), no contrast or structure is visible except for the adipose tissue, indicated by a blue arrow in the top right section. This is due to the homogeneous structure of the fibroadenoma. Since its deviation in attenuation is very small compared to the surrounding tissue, it cannot be resolved in the attenuation images.

The high-dose phase-contrast image reveals a cell-rich region in the center of the fibroadenoma, indicated by a red arrow, cf. Fig. 4(c). This region is visible in the low-dose phase-contrast image, although the noise level is higher, whereas it is not in the attenuation-contrast images. The CNR values in Table 2 calculated for this cell-rich region marked in Fig.4(c) support this observation. The CNR values for the attenuation-contrast images are below one, indicating only noise in the region. In contrast the CNR values for the phase-contrast images are more than ten times higher.

**Table 2:** CNR analysis of the fibroadenoma. The CNR of the phase-contrast images reveal a significantly increased CNR compared to the attenuation images. Both image modalities are obtained from the same dataset with gratings in the beam, resulting in approximately double the dose compared to a scan without gratings.

Modality	${26}\,\hbox {mGy}$	${9}\,\hbox {mGy}$
Attenuation	0.26	0.13
Phase Contrast	3.31	1.91

## Discussion and conclusion

We have demonstrated that gbpc-BCT significantly enhances the visual contrast and CNR compared to an attenuation-based BCT. Therefore we measured a breast phantom and a freshly dissected fibroadenoma at a laboratory gbpc-BCT setup with a quasi-monoenergetic X-ray source. Thereby the achieved MGD of ${10}\,\hbox {mGy}$ is only slightly higher than the one of the commercial BCT devices used in clinics^7^. However, because the method offers three complementary image modalities, namely attenuation, phase contrast and dark field, and consequently a drastic information gain compared to a conventional BCT, the additional dose might be justified for diagnostic examinations, yet further evaluation is needed to prove this.

The phase contrast and attenuation images shown here were obtained from the same measurements with gratings in the beam. The analyzer grating between the sample and detector absorbs about half the radiation dose, resulting in almost twice the MGD compared to a BCT measurement without gratings. However, the phase-contrast image of the fibroadenoma measurement at ${9}\,\hbox {mGy}$ shows a CNR of 1.91, whereas the attenuation channel at 2.8 times the dose has only a CNR of 0.26. Therefore, despite the attenuation image receiving more than twice the dose, the phase-contrast image still exhibits a significantly higher CNR.

The dark-field image is beneficial for detecting and classifying microcalcifications and discriminating between benign and malignant cysts^34,39^, but was neglected in this work because the samples did not contain small-angle scattering structures such as calcifications, and the focus was on extracting the phase information in a low-dose gbpc-BCT.

Nevertheless, the dose remains a challenge which needs to be tackled in order to get the method closer to a clinical practice. The MGD for BCT scans is often calculated with special DgN_CT_values, accounting for the rounded shape of the uncompressed breast^40^. However, since these factors have not been simulated for the required volume and diameter, we used the DgN factors in our study. These factors overestimate the volume and, as a result, the dose. Therefore, the calculated dose represents an upper limit of the actual dose administered due to the exaggerated volume of the sample. Furthermore, we had limited time to scan our clinical samples, because of the need for prompt histological analysis. This resulted in samples with a maximum diameter of ${2.5}\,\hbox {cm}$, which is significantly smaller than the average breast size. While smaller samples are more dose-efficient, achieving comparable imaging quality in a complete breast would require a higher radiation dose. Additionally, the measurements were conducted using a quasi-monochromatic X-ray source to assess the maximum benefit of gbpc-BCT as a preliminary proof of concept before transitioning to a more challenging polychromatic X-ray source. In particular, for measurements with a polychromatic X-ray source, the efficiency of the interferometer decreases compared to the monoenergetic source. However, Rawlik et al. demonstrated that due to its increased soft tissue contrast a gbpc-BCT can even be more dose-efficient than a conventional BCT^41^.

Furthermore, self-supervised convolutional denoising algorithms like Noise2Inverse^42^,which are in already well tested with conventional CT, reduce the MGD while maintaining the CNR, to get closer to a clinically relevant dose. On the other hand, more advanced reconstruction techniques have to be applied, such as intensity-based statistical iterative reconstruction, which requires just one single image per angular position to retrieve the phase and attenuation information^43^.

In summary, gbpc-BCT combines the advantages of phase contrast imaging and BCT, namely, higher soft tissue contrast, no superimposed tissue structures, and higher patient comfort. Overall, it is a promising solution for breast cancer detection and diagnosis and could, in the future, allow to reduce the dose for X-ray based breast examinations.

## Data Availability

The data that support the findings of this study are available from the corresponding author on reasonable request.
